# One Score Fits All? A Narrative Review on Early Warning Scores for Older Adults in the Emergency Department in the Era of Personalized Medicine

**DOI:** 10.3390/jpm16020098

**Published:** 2026-02-06

**Authors:** Valeria Maccauro, Piergiacomo Maria Cacciamani Fanelli, Davide Antonio Della Polla, Nicola Bonadia, Giuseppe De Matteis, Andrea Piccioni, Antonio Gasbarrini, Claudio Sandroni, Francesco Franceschi, Marcello Covino

**Affiliations:** 1 Emergency Department, Fondazione Policlinico Universitario A. Gemelli IRCCS, 00168 Rome, Italy; 2 Department of Geriatrics, Fondazione Policlinico Universitario A. Gemelli IRCCS, 00168 Rome, Italy; 3 Department of Internal Medicine and Gastroenterology, Fondazione Policlinico Universitario A. Gemelli IRCCS, 00168 Rome, Italy; 4 Università Cattolica del Sacro Cuore, 00168 Rome, Italy; 5 Department of Anesthesiology and Intensive Care Medicine, Fondazione Policlinico Universitario A. Gemelli IRCCS, 00168 Rome, Italy

**Keywords:** early warning scores, older adults, emergency department, frailty, risk stratification, personalized medicine

## Abstract

**Background:** The growing use of Emergency Departments (EDs) by older adults highlights the need for early and accurate identification of clinical deterioration. Early Warning Scores (EWSs) are widely implemented tools based on standardized vital sign thresholds; however, their performance in elderly patients is inconsistent, likely reflecting the biological heterogeneity, multimorbidity, and reduced physiological reserve typical of this population. **Objectives:** This narrative review aims to summarize current evidence on the use of EWSs in adults aged ≥ 65 years presenting to the ED, with a specific focus on mortality and intensive care unit (ICU) admission, and to discuss their role within the evolving framework of personalized medicine. **Sources:** A narrative review of 36 clinical studies published between 2014 and 2025 was conducted. **Content:** Traditional scores such as National Early Warning Score (NEWS), National Early Warning Score 2 (NEWS2), Modified Early Warning Score (MEWS), VitalPAC Early Warning Score (ViEWS), Rapid Acute Physiology Score (RAPS) and Rapid Emergency Medicine Score (REMS) show variable and often reduced prognostic accuracy in older and frail patients. Evidence consistently suggests that applying uniform cut-off values fails to capture individual vulnerability in elderly patients. The integration of age, frailty, comorbidities, and baseline physiological status improves risk stratification. Second-generation tools—including Copeptin-NEWS, NEWS-L, suPAR-NEWS, OPERA, and RISE UP—as well as artificial intelligence-based models, represent emerging personalized approaches to clinical deterioration prediction. **Implications:** No single score currently provides reliable early risk prediction for all elderly ED patients. Moving beyond “one-size-fits-all” EWSs toward adaptive, person-centered models may better reflect the complexity of geriatric emergency care and improve prognostic accuracy.

## 1. Introduction

The global rise in life expectancy and the demographic shift toward aging populations have led to a substantial increase in emergency department (ED) utilization by older adults. In the United States, individuals aged 65 years and older accounted for approximately 20% of all ED visits in 2022, totaling over 33 million consultations [1]. Similar trends are observed across Europe, where older adults represent the fastest-growing patient group in emergency settings [2].

This demographic transition challenges traditional “one-size-fits-all” approaches to emergency risk stratification, as older adults constitute a highly heterogeneous population in terms of functional status, comorbidity burden, baseline physiological parameters, and biological resilience.

Older patients frequently present with complex clinical profiles characterized by multiple chronic diseases, polypharmacy, cognitive impairment, functional decline, and atypical or non-specific symptom presentations [3,4,5,6,7]. These factors complicate diagnostic accuracy and timely decision-making in the ED.

Moreover, the intrinsic vulnerability of older adults is exacerbated by the stressful and overcrowded ED environment, as well as by acute illness itself. Reduced physiological reserve may lead to rapid clinical deterioration, often preceded by subtle or atypical signs. Altered mental status, frequently coexisting with comorbidities, further complicates early recognition of clinical instability [8,9,10].

In the general adult population, Early Warning Scores (EWSs) are widely adopted to detect early signs of deterioration and prompt escalation of care. These tools generate composite scores based on deviations of vital signs from predefined thresholds, supporting standardized clinical decision-making [11,12]. However, their reliance on fixed cut-off values may conflict with the principles of personalized medicine when applied to elderly patients, whose baseline physiology and responses to acute stressors vary widely.

Indeed, the performance of EWSs in older adults remains insufficiently validated. Chronic diseases and pharmacological treatments may blunt or mask physiological responses, leading to underestimation of risk and delayed intervention [13,14,15]. Older patients experience higher rates of unexpected deterioration and mortality during ED stay compared with younger individuals [16]. Notably, incorporating age into standard EWSs partially mitigates this underestimation, particularly in patients over 80 years of age [15].

Traditional triage tools such as the Emergency Severity Index (ESI) identify fewer than half of older adults requiring life-saving interventions [17]. Similarly, geriatric-specific screening tools such as the Identification of Seniors at Risk (ISAR) and the Triage Risk Stratification Tool (TRST) have demonstrated limited standalone predictive value [18,19].

Taken together, these findings suggest that EWSs should be re-evaluated not only as predictive tools, but also as components of a broader, individualized clinical decision-making framework. The aim of this review is to provide a comprehensive overview of current evidence regarding the use of EWSs in older adults in the ED, through the lens of personalized medicine.

## 2. Material and Methods

We conducted a narrative review of the current literature available on Pubmed, Scopus and Elsevier: we examined all the clinical trials conducted among the role of EWS in predicting mortality and intensive care unit (ICU) admission in elderly (>65 years old) people admitted to ED between 2014 and 2025. For our research, we selected the following key words: (early warning scores) AND (elderly) AND (emergency department) AND (mortality). The majority of studies examined were observational trials, both prospective and retrospective (see Table 1 and Table 2). The literature search was completed in October 2025.

Studies published before 2014, non-English articles, and trials involving populations younger than 65 years were excluded. A total of 36 clinical studies, screened for population >65 years old, emergency department setting and the presence of at least one EWSs as a predictor of mortality or ICU admission, were selected and examined to find out the more suitable score to predict adverse outcome in elderly critical ill patients: in fact, Table 1 and Table 2 represented a synthesis of the principal outcomes and limits of each article, while in the following 4 sections a critical discussion of each trial was performed to take the appropriate conclusions. All these studies were selected by one of the authors of the paper.

## 3. Traditional EWS

Traditional EWSs have been validated in general adult populations; however, their effectiveness in older adults remains controversial [16,20,21,33,52,53,54]. This section summarizes the most commonly used EWSs, with a focus on their applicability to elderly ED patients.

### 3.1. News

The National Early Warning Score (NEWS) evaluates clinical vital signs, including respiratory rate (RR), oxygen saturation, systolic blood pressure, heart rate (HR), level of consciousness or altered mental status and body temperature. Based on the complexity point, patients are classified into three categories (low risk, NEWS 1–4, medium risk NEWS 5–6 and high risk NEWS ≥7) and treated in different hospital setting from medical ward to intensive care unit, based on the degree of the deterioration risk [55].

In its original formulation, age was deliberately excluded from the National Early Warning Score (NEWS), despite being a well-established independent predictor of mortality. The rationale cited was the minimal improvement observed in the area under the receiver operating characteristic (AUROC) curve; however, this justification failed to consider other relevant performance metrics, such as calibration. Moreover, age was treated as a dichotomous variable (≥65 years), potentially limiting its predictive contribution.

NEWS has been used to identify early cardiac arrest, unanticipated ICU admission [56], acute medical admissions [57] and non-elective surgical admissions [58] even in prehospital settings [59]. In fact, among patients at risk of adverse clinical outcomes, the NEWS had an equivalent performance in identifying patients similar to the Medical Emergency Team Criteria (MET) [60,61].

NEWS has also been reported to be capable of predicting the deterioration of patients in the ED with sepsis, with an overall equivalent or even superior value for Quick Sequential Organ Failure Assessment (qSOFA) and Systemic Inflammatory Response Syndrome (SIRS) criteria in predicting mortality and ICU admission [62].

In 2019 a single-center retrospective study firstly demonstrated that an elevated NEWS score (cut-off 5 points) at the admission to ED was significantly correlated with in-hospital mortality in geriatric patients over 65 years old, with an increasing rate of approximately 47.5% for each 1 point raising [27]. Nevertheless, no comparison was made with younger adults regarding the effectiveness of such predicting score. Furtherly, following evidence observed that, comparing with younger patients, in older people NEWS did not show the same prognostic performance, thus providing only a moderate accuracy in predicting mortality [16,54].

### 3.2. News 2

The NEWS2 score includes assessment of consciousness, systolic blood pressure, respiratory rate, body temperature, heart rate, need for supplemental oxygen, and re-evaluation after oxygen administration, making it a potentially valuable tool for older adults with type II respiratory failure [25].

However, a recent study reported that NEWS2 has lower accuracy in predicting 30-day mortality in frail older adults (>75 years) compared to younger patients, with an AUC of 0.70 (95% CI: 0.64–0.76) versus 0.88 (95% CI: 0.85–0.90) [20]. This highlights the need for improved methods to enhance NEWS2 performance in the elderly subgroup.

One proposed modification involves the inclusion of relative hypotension, defined as a drop in systolic blood pressure >7 mmHg compared to baseline values from primary care records. This adjustment has been shown to marginally improve NEWS2’s predictive accuracy for 30-day mortality in older adults [63].

Furthermore, a recent observational study involving geriatric patients with respiratory failure and delirium hypothesized that combining delirium with respiratory parameters and a modified version of NEWS2—excluding the “Oxygen Saturation” and “Any Supplemental Oxygen” components to better capture non-hypoxemic acute illness—could yield a more comprehensive score for predicting 30-day mortality in the ED (OR: 2.26; 95% CI: 1.08–4.72) [64]. A separate retrospective study also found that adding delirium to NEWS2 increased alert levels in the ED [65].

Nevertheless, further research is needed to validate these modified tools in specific geriatric subgroups [64].

## 4. MEWS

The Modified Early Warning Score (MEWS) is one of the most widely used EWS tools at the time of emergency department (ED) admission. It is based on five physiological parameters: systolic blood pressure, heart rate, respiratory rate, body temperature, and neurological status [66]. MEWS has been positively correlated with both ICU admission rates and mortality in multiple clinical studies [67,68].

Notably, dynamic changes in MEWS—rather than a single measurement—have been associated with an increased risk of adverse outcomes [46,69,70]. This concept was already highlighted in a 2017 single-center study, which found that worsening respiratory rate and heart rate were linked to rising MEWS scores and overall clinical deterioration. Interestingly, the study also observed that vital sign derangements occurred more frequently in older patients compared to younger ones, suggesting that age may act as an additional risk factor for increased mortality [71].

### Other Clinical EWS

The VitalPac Early Warning Score (ViEWS) incorporates systolic blood pressure, heart rate, respiratory rate, temperature, oxygen saturation, use of inhaled oxygen, and the AVPU (Alert, Verbal, Pain, Unresponsive) scale [48]. Similarly to MEWS, its predictive ability for mortality has been reported to improve when an additional point is allocated to patients aged ≥65 years [14].

In parallel, the Rapid Acute Physiology Score (RAPS) evaluates heart rate, mean arterial pressure, respiratory rate, and Glasgow Coma Scale (GCS) [72], while the Rapid Emergency Medicine Score (REMS) includes age, heart rate, mean arterial pressure, respiratory rate, GCS, and peripheral oxygen saturation [73]. Notably, a retrospective analysis conducted in 2019 found that REMS demonstrated superior accuracy in predicting mortality among adult ED patients, outperforming both MEWS and RAPS [74]. A possible explanation for this superiority is the inclusion of age in REMS, which is absent in several other EWSs and may represent an independent risk factor for in-hospital mortality [74].

Nevertheless, specific studies validating REMS as a predictor of mortality in the elderly population are still lacking. Overall, traditional EWSs tend to underestimate the risk of deterioration in older patients, suggesting that age itself may be an additional factor to consider—although its exact role in modifying the predictive performance of EWSs remains unclear [33].

Indeed, applying the same generic vital sign thresholds across all age groups, without accounting for individual baseline values, may result in missed cases of clinical decompensation. For example, older adults often present with chronic hypertension, which may mask a significant drop in systolic pressure that would otherwise indicate deterioration. Conversely, they may score fewer points for heart rate due to an impaired cardiovascular response to stress. Similarly, older patients are less likely to exhibit fever or elevated respiratory rates, resulting in lower EWS scores compared to younger individuals [75].

Given these considerations, there is a compelling need to develop specific predictive and prognostic scoring systems tailored to elderly patients, incorporating age-related physiological patterns, comorbidities, and frailty.

## 5. Prediction of Mortality

Since 2014, a total of 29 clinical studies have investigated the role of Early Warning Scores (EWSs) in predicting mortality among older adults. (Table 1)

An observational study published in 2015 demonstrated that the 7-day mortality rate estimated using EWSs at ED admission was significantly higher in older patients (aged 60–79 and >80 years) compared to younger individuals, regardless of the score used [33]. Importantly, after adjusting for comorbidities, the mortality difference between the 60–79 age group and younger patients was attenuated, while the >80 age group maintained a statistically significant elevated mortality rate [33].

This study laid the foundation for the hypothesis that age may act as an independent risk factor for mortality, beyond the predictive capacity of traditional EWSs. Subsequent research has focused on the impact of age in modifying the prognostic performance of these scores.

In 2022, a multicenter retrospective study conducted in Denmark found that the NEWS score underestimated mortality risk in patients over 80 years, with age emerging as the strongest predictor of in-hospital mortality (AUROC 0.82; 95% CI: 0.80–0.84) [15]. One possible explanation is that the association between vital sign derangement and mortality is stronger in older adults, or that vital sign abnormalities alone are insufficient to predict mortality in this population, likely due to additional factors such as frailty [13]. Notably, combining NEWS with age improved its predictive performance for in-hospital mortality [15], although this enhancement did not extend to ICU admission rates—possibly because older patients are less frequently candidates for intensive care due to comorbid conditions [15].

Building on this hypothesis, a multicenter prospective study demonstrated that incorporating age into traditional EWSs improved their ability to predict mortality, with NEWS2 showing the highest performance. Mortality was notably higher in patients aged ≥75 years, with a marked increase observed from age 80 onwards [28]. This finding aligns with the understanding that physiological parameters included in EWSs are significantly influenced by aging. For example, progressive reductions in lung capacity may lead to compensatory increases in respiratory rate, while age-related conditions such as hypertension, bradycardia, COPD, atrial fibrillation, stroke, and dementia can alter the reliability of vital signs used in EWSs [76].

In 2023, an international multicenter cohort study conducted in the Netherlands developed the International Early Warning Score (IEWS), a recalibrated version of NEWS that incorporates age and sex. The score was evaluated across three age categories (18–65, 66–80, >80 years) at ED arrival. The IEWS outperformed NEWS in predicting in-hospital mortality across all age groups (AUROC 0.89 [95% CI: 0.89–0.90] vs. 0.82 [95% CI: 0.82–0.83]) [21], suggesting that this new score may overcome the limitations of traditional EWSs in older adults.

A prospective observational study conducted in a Finnish ED compared NEWS2 with a local three-level triage scale in patients aged ≥75 years. Both tools were statistically significant predictors of 30-day mortality and ICU admission (*p* < 0.001), but showed poor accuracy in predicting ED length of stay and readmission rates [20].

Another retrospective observational study compared NEWS and MEWS at ED admission in patients aged >65 years. NEWS demonstrated superior predictive accuracy for in-hospital mortality (AUROC 0.789 vs. 0.720; *p* < 0.001), with optimal cut-off values of >5 and >4 points, respectively [10].

Additionally, a retrospective study involving 651 geriatric patients evaluated the performance of NEWS2 versus the Laboratory Data Decision Tree Early Warning Score (LDT-EWS), which incorporates laboratory parameters such as blood urea nitrogen (BUN), creatinine, sodium, potassium, white blood cell count, hemoglobin, and albumin [26]. The combination of NEWS2 and LDT-EWS yielded the highest predictive accuracy for in-hospital mortality [26]. Notably, the LDT-EWS had previously been validated in a 2015 retrospective study as a reliable predictor of mortality in patients over 75 years admitted to medical wards [77].

In contrast, another study found that NEWS2 alone had limited ability to predict 30-day mortality in geriatric patients, while the addition of age significantly improved its discriminative power [36].

## 6. Prediction of Mortality Under Specific Clinical Conditions

To address the limitations of traditional EWSs in elderly populations, several new scoring systems have been proposed to identify geriatric patients at risk of mortality under specific clinical conditions (Table 1).

For instance, a retrospective cohort study involving 599 geriatric patients with suspected sepsis (mean age: 77 years) developed the Ramathibodi Older Sepsis Score (ROSS) to predict 28-day mortality at ED admission. The score ranges from 0 to 15 points, with a cut-off ≥6 identifying moderate-to-high risk patients requiring rapid transfer to inpatient wards or ICU. Variables such as malignancy, dependent status, heart rate (≤49 bpm or ≥120 bpm), respiratory rate (≥24 bpm), oxygen saturation ≤93%, altered consciousness, and lactate ≥4 mmol/L demonstrated excellent discriminative ability (AUROC: 0.87; 95% CI: 0.82–0.92), outperforming both NEWS (AUROC: 0.74) and REWS (AUROC: 0.71) (*p* < 0.01) [22].

Another specific condition is non-traumatic coma (NTC), defined as coma or altered mental status due to non-traumatic causes. A retrospective study of 683 geriatric patients compared the predictive accuracy of NEWS and MEWS for in-hospital mortality in NTC. Both scores were significantly higher in non-survivors compared to survivors (NEWS: 10 [8,9,10,11,12] vs. 7 [5,6,7,8,9,10]; MEWS: 7 [5,6,7,8] vs. 5 [4,5,6]), but only NEWS (cut-off > 7) showed statistically significant predictive value (adjusted OR: 1.323 vs. 1.253) [24].

In 2022, an observational study demonstrated that MEWS was directly correlated with worse prognosis and increasing New York Heart Association (NYHA) class in patients >60 years with chronic heart failure (CHF) (*p* < 0.05) [78].

A recent retrospective study applied EWSs to detect post-operative clinical deterioration. A comparison between patients younger and older than 70 years revealed no statistically significant difference in identifying post-operative complications using a threshold score of ≥3 points [79].

Moreover, a prospective study of patients >65 years with acute upper gastrointestinal bleeding compared the effectiveness of RAPS, REMS, and MEWS in predicting in-hospital mortality. MEWS demonstrated the best performance, with a cut-off >2 points, including in the very elderly subgroup (>75 years) [31].

Finally, a retrospective study of elderly patients with COVID-19 infection found that none of the evaluated EWSs (REMS, RAPS, MEWS) were statistically effective in predicting mortality [32]. Another clinical study conducted by Covino et al. among elderly patients with COVID 19 observed, in contrary, that NEWS performance in predicting mortality was similar to other specific scores [International Severe Acute Respiratory Infection Consortium Clinical Characterization Protocol Coronavirus Clinical Characterization Consortium (ISARIC-4C) score, COVID-GRAM Critical Illness Risk Score (COVID-GRAM), quick COVID-19 Severity Index (qCSI)] [AUROC 0.764 (0.700–0.819), *p* < 0.001, vs. 0.799 (0.738–0.851 for the ISARIC-4C, 0.785 (0.723–0.838) for the COVID GRAM, 0.749 (0.685–0.806) for the qCSI)] [44]. This finding contrasts with previous evidence showing REMS as a reliable predictor of 7-day poor outcomes in adult patients with SARS-CoV-2 infection. A possible explanation is that the age component, which enhances REMS performance in the general population, loses discriminative power in elderly patients (>65 years), who share similar age-related risk factors [80].

## 7. Prediction of Intensive Care Unit Admission

Different clinical studies have explored the association between Early Warning Scores (EWSs) and ICU admission in older adults presenting to the emergency department (ED) (Table 2); however, none of the evaluated scores has consistently demonstrated statistically superior performance.

In 2015, a prospective single-center observational study compared the effectiveness of MEWS and ViEWS in predicting ICU admission and mortality in geriatric patients (>65 years) admitted to the ED. The authors found no statistically significant difference between the two scores, using cut-off values of 4 for MEWS and 8 for ViEWS, respectively [48].

In 2019, a retrospective cohort study involving older patients at high risk of deterioration showed that NEWS, with a cut-off of 9 points, was effective in predicting ICU admission, ICU mortality, and in-hospital mortality, with no significant differences observed between patients aged above and below 80 years [49].

Similarly, a retrospective cohort study of 409 older patients (mean age: 79.5 years) with influenza demonstrated that a NEWS score ≥8 was significantly associated with ICU admission [OR: 5.37; 95% CI: 2.61–11.04], regardless of influenza type A or B [45].

During the COVID-19 pandemic, a retrospective study compared the predictive performance of NEWS, qSOFA, the Charlson Comorbidity Index (CCI), and the Elixhauser Comorbidity Index (ECI) in elderly patients admitted to the ED [39]. Although older adults were known to have a higher mortality risk due to comorbidities during the pandemic [81], the study found that NEWS was not a reliable predictor of mortality in this subgroup. The reasons for this limited performance remain unclear [39].

A retrospective cohort study of non-traumatic elderly ED patients (≥65 years) admitted to the ICU found that the trend in Emergency Department Modified Early Warning Score (EDMEWS) progression was significantly associated with 30-day mortality, independent of ED length of stay [46].

In 2021, a comprehensive retrospective analysis focusing on elderly patients with pneumonia reported that MEWS (cut-off ≥ 4) had the best performance in predicting mortality [AUC: 0.927; *p* < 0.001], while NEWS (cut-off ≥ 5) was superior in predicting ICU admission [AUC: 0.976; *p* < 0.001] [50].

Most recently, in 2025, a retrospective study compared REMS and NEWS2 in predicting ICU admission and mortality among older (>65 years) and younger (18–64 years) ED patients. The study found that REMS performance declined in the older cohort for both endpoints, while NEWS2 showed reduced predictive value only for in-hospital mortality in older adults [51]. Additionally, the optimal cut-off values differed substantially between age groups, particularly for REMS (5–6 in adults vs. 10 in older adults), suggesting that EWSs may require age-specific calibration or the integration of geriatric-specific tools to optimize their use in older populations [51].

## 8. The Variable of Frailty

Frailty represents a multidimensional construct reflecting reduced physiological reserve and increased vulnerability to stressors [82]. Approximately 40% of older adults presenting to EDs in Europe are considered frail [2], with frailty independently associated with early mortality and adverse outcomes [83,84].

From a personalized medicine perspective, frailty captures dimensions of risk not reflected by vital signs alone, including cognition, mobility, functional status, and social vulnerability. These factors may explain why frail patients experience adverse outcomes even in the absence of marked physiological derangements.

Frailty has been independently associated with mortality both at ED presentation and within 72 h of admission [84]. Given that frailty encompasses impairments in cognition, mobility, functional status, and comorbidities, it has been hypothesized that frail patients may be less able to tolerate severe vital sign derangements as measured by traditional EWSs [47]. In particular, delirium and cognitive decline have emerged as strong predictors of mortality and treatment limitations [47]. However, the clinical identification of frailty remains challenging in the ED setting due to the subtle and often nonspecific nature of its signs and symptoms [85].

Several studies have proposed combining frailty, vital signs, and overall illness or injury severity into a composite “geriatric urgency score” to better identify older adults at high risk of deterioration and mortality in the ED [86]. Frailty is commonly assessed in inpatient settings using subjective tools such as the Clinical Frailty Scale (CFS), which ranges from 1 to 9, with a score ≥6 indicating severe frailty [87]. Nevertheless, standardized and objective tools for measuring frailty have not yet been validated for use in critically ill ED patients [88].

An observational study of 109 patients with NEWS ≥ 7 found that frail individuals had higher ICU admission rates within 90 days and increased 90-day mortality compared to non-frail patients (46% vs. 27%; adjusted OR: 1.95; 95% CI: 0.84–4.55) [47]. The authors also noted a higher prevalence of frailty among patients with severe vital sign abnormalities, and that treatment limitations outside the ICU were more frequently applied to frail patients [47].

Another observational cohort study evaluated the impact of comorbidities on EWS performance in predicting 7-day mortality. The study showed that among patients with low acuity (EWS 0–4), a Charlson Comorbidity Index (CCI) score ≥ 3 significantly increased mortality risk, regardless of EWS value. In contrast, for patients with higher acuity (EWS ≥ 5), CCI had no significant impact after adjusting for age [89]. Notably, the inclusion of age in EWS systems has previously been shown to enhance predictive accuracy for both in-hospital mortality and ICU admission [90].

In addition, a study involving 500 older patients with altered mental status admitted to the ED demonstrated that NEWS2 was a better predictor of mortality than qSOFA (AUROC: 0.725; 95% CI: 0.683–0.763 vs. 0.631; 95% CI: 0.587–0.673), with an optimal cut-off >5 points [25].

A recent retrospective study found that the Frailty Index–Laboratory (FI-lab), derived from routine blood test results, had comparable accuracy to NEWS in predicting in-hospital mortality among patients aged > 65 years, with a cut-off value of 0.52 [30].

Taken together, these findings suggest that combining frailty assessments with traditional EWSs may help overcome current limitations in predicting mortality among older ED patients. For example, the Frailty-adjusted Prognosis in the Emergency Department (FaP-ED), which integrates NEWS and CFS, has demonstrated superior predictive performance compared to either tool alone in estimating 30-day mortality [34]. Moreover, frailty assessment has been proposed as a useful predictor of broader adverse outcomes, including ED readmission rates, although standardized methods for objectively quantifying clinical dysfunction are still needed [84].

Nevertheless, further clinical studies are required before frailty can be routinely incorporated into traditional EWSs in everyday ED practice.

## 9. New Prospectives

Emerging biomarkers, composite scores, and artificial intelligence–based models aim to overcome the intrinsic limitations of standardized EWSs by incorporating individualized biological, laboratory, and contextual data.

These approaches align with precision-oriented emergency medicine, allowing dynamic risk estimation rather than static threshold-based classification.

To address the limitations of traditional Early Warning Scores (EWSs) in predicting mortality and ICU admission among elderly patients, several novel biomarkers and composite scoring systems have been investigated to enhance risk stratification in the emergency department (ED).

Copeptin, a 39-amino acid glycopeptide derived from the C-terminal portion of pre-provasopressin (pre-proAVP), is released alongside vasopressin (AVP) and reflects AVP’s physiological role in maintaining blood pressure, intravascular volume, and plasma osmolality under stress conditions such as acute illness. Given the established association between AVP and increased mortality, copeptin has emerged as a promising biomarker for mortality prediction [91,92]. A recent prospective study demonstrated that the combination of copeptin > 19.78 pg/mL and NEWS > 8.5 significantly improved the prediction of 30-day mortality in critically ill patients aged > 60 years (*p* < 0.05) [23].

In a multicenter prospective cohort study involving 687 older patients (median age: 79 years), the addition of end-tidal carbon dioxide (ETCO_2_) and perfusion index (PI) to the NEWS2 score enhanced its predictive accuracy for 30-day in-hospital mortality [12]. Both ETCO_2_ and PI are routinely measured at the bedside using non-invasive devices during cardiopulmonary resuscitation and are considered indirect indicators of cardiac output. ETCO_2_ reflects ventilation and metabolic status, while PI assesses peripheral perfusion and vascular tone; lower values of both have been associated with increased mortality risk [93,94].

Lactate has also been explored as an adjunct to EWSs. A retrospective study in geriatric patients (>65 years) found that combining lactate levels with NEWS (NEWS-L score) improved the prediction of in-hospital mortality [29]. Lactate is a widely used biomarker in critical care settings, including sepsis, hemorrhagic shock, and trauma [95]. However, the absence of a standardized lactate threshold limits its utility in guiding clinical decisions [29]. Contrarily, a prospective study failed to demonstrate any improvement in predictive accuracy when lactate was added to MEWS and NEWS in elderly patients [35]. Nonetheless, another retrospective study showed that incorporating lactate into ViEWS significantly enhanced its predictive performance (AUROC: 0.872) [38], indicating that the role of lactate in geriatric populations remains controversial.

Soluble urokinase plasminogen activator receptor (suPAR) has been proposed as another novel biomarker. A prospective Japanese study found that combining suPAR with NEWS (suPAR-NEWS) significantly improved predictive accuracy compared to either score alone [42].

In parallel, a comprehensive clinical score named OPERA (Older Persons’ Emergency Risk Assessment) was developed to predict in-hospital mortality in elderly ED patients. This model integrates NEWS2, the Clinical Frailty Scale (CFS), the Malnutrition Universal Screening Tool, acute kidney injury, age, and sex. OPERA demonstrated superior predictive performance compared to individual scores [37].

Another emerging tool is the Respiratory Adjusted Shock Index (RASI), which combines the traditional shock index (heart rate/systolic blood pressure) with respiratory rate. RASI has shown positive correlation with NEWS2 in geriatric patients [40] and has been successfully used to predict outcomes in adult patients with sepsis and trauma [96,97].

The Prehospital Drug-Derived Score (PDDS), based on medications administered prior to ED arrival, has demonstrated comparable accuracy to NEWS2, RAPS, and REMS in predicting mortality among patients aged >75 years. Its performance may be attributed to the inclusion of age as a parameter [41].

A European multicenter study developed the RISE UP score, composed of six variables: age, ≥2 abnormal vital signs, serum albumin, blood urea nitrogen, lactate dehydrogenase, and bilirubin. This score outperformed MEWS in predicting 30-day mortality in elderly ED patients [43]. However, further validation is required before these comprehensive scores can be routinely implemented in clinical practice.

Artificial intelligence (AI) has recently been explored as a means to overcome the limitations of traditional EWSs. In 2022, a cohort study of hospitalized elderly patients (median age: 76.1 years) demonstrated that the Epic Deterioration Index (EDI), a machine learning-based model, was associated with a 10.4 percentage point absolute reduction in care escalation events, including ICU transfer and in-hospital death (95% CI: −20.1 to −0.8; *p* = 0.03) [98].

Furthermore, a retrospective cohort study involving 362,962 patients (median age: 64 years) found that eCARTv5, a gradient-boosted machine learning model, was the most accurate tool for identifying clinical deterioration (AUROC: 0.895), outperforming other AI-based models such as EDI and the Rothman Index (RI), as well as traditional EWSs including MEWS, NEWS, and NEWS2. Notably, NEWS was the second-best performer (AUROC: 0.831), surpassing both EDI and RI [99].

## 10. Limitations

Despite our intention to provide a solid scientific statement, the most relevant limitation of this paper is its narrative nature. Nevertheless, we hope to ha posed the basis for future randomized clinical trials comparing the different aforementioned EWSs either with each other or with the new developed ones. Another important limitation is that the majority of the studies examined are observational trials, with limited intrinsic statistical significance. Moreover, the heterogeneity of study designs and population’s age may limit their direct comparison. Therefore, the need of randomized clinical studies among the predictive role of EWSs in terms of mortality and ICU admission in elderly patients, with a clear distinction in different age subgroups, is still an open issue.

## 11. Conclusions

Despite ongoing advancements, there is currently no simple, rapid, and reliable tool capable of accurately predicting early clinical deterioration in critically ill elderly patients [10], as the choice of a specific scoring system should be guided by the clinical context.

Although NEWS and NEWS2 have been frequently reported as effective predictors of mortality and ICU admission in older adults admitted to the ED for various medical conditions [10,20,24,45,49,99], the majority of current evidence emphasizes the need to enhance these scores by incorporating age and frailty as additional variables [13,15,33,34].

In this context, several clinical and laboratory-based tools are under validation to develop second-generation EWSs, such as Copeptin-NEWS [23], NEWS-L [29], ViEWS-L [38], suPAR-NEWS [42], and OPERA [37]. Additionally, innovative scoring systems including PDDS [41], RISE UP [43], and AI-based models [98,99] have been proposed to improve risk estimation in elderly ED patients.

Nevertheless, while traditional validated tools continue to be employed in clinical practice, their reduced accuracy in prognostic predictions for older adults highlights the need to move beyond rigid, guideline-based approaches. A shift toward adaptive, person-centered decision-making frameworks is warranted, ones that integrate frailty indices, functional status assessments, and multimorbidity profiles to better reflect the complexity of geriatric patients [51].

In this regard, it might be reasonable considering the new data-driven artificial intelligence platforms as the solution for the question of the comprehensive stratification risk system for geriatric patients: in fact, as big data systems could involve a large amount of physiological information for each patient, they might powerfully drive clinical decisions even in comorbid subjects. However, as explained in the aforementioned study among eCARTv5 [99], the slight outperforming of clinically derived scores requires large datasets and substantial computational resources, yet the most influential predictors remain unchanged. In addition, it could be questionable if the replacement of the human clinical judgment in terms of risk stratifications with preformed rigid data sets would really be the solution to avoid the actually unpredicted events and to obtain a better resource allocation. Notably, in real-life ED setting, older patients identified as at high risk of deterioration by scoring systems are often not eligible to intensive supports due to their clinical general status and to the limited resources. Therefore, in valuating each EWS for clinical applicability in older patients in an ED setting we should answer the following question: do score sensitivity and precision value really reflect an operative resource allocation or are still activating a low-yield alarm rate? At this time, this is still an open issue.

## Figures and Tables

**Table 1:** Clinical trials among the role of EWSs in predicting mortality in elderly patients admitted to ED.

Kemp, K. et al. 2020 [20]	prospective, observational, monocentric study N = 1711 (mean age 85 years)	2018–2019	NEWS2 vs. 3-level triage scale	Lower accuracy in predicting mortality than in other studies with younger patients. NEWS2 and triage score, AUCs for 30-day mortality prediction were 0.70 (0.64–0.76, *p* < 0.001) and 0.62 (0.56–0.68) respectively AUCs for ICU admission were 0.72 (0.61–0.83, *p* < 0.001) for NEWS2 vs. 0.80 (0.70–0.90) for 3-leve l triage scale
Candel, B.G.J. et al. 2023 [21]	International multicenter cohort study N = 95,553	2017–2019	IEWS vs. NEWS in patients admitted to ED (18–65 years, 66–80 years, >80 years)	IEWS performed significantly better than NEWS (AUC 0.89 [95% CI, 0.89–0.90] vs. 0.82 [0.82–0.83]) in predicting in-hospital mortality in all age categories
Sanguanwit, P. et al. 2024 [22]	Retrospective monocentric cohort study N = 599 (mean age 77 years)	2018	Geriatric patients admitted to ED with suspected sepsis: ROSS score	ROSS was better in prediction 28-day mortality [AUROC: 0.87, 95% confidence interval (CI): 0.82–0.92)] than qSOFA (AUROC: 0.72), NEWS (AUROC: 0.74), and REWS (AUROC: 0.71) (*p* < 0.01)
Wang, F. et al. 2021 [23]	Prospective monocentric observational study N = 205 (>60 years)	2017–2018	Copeptin + NEWS vs. copeptin and NEWS individually in predicting mortality in elderly patients with critical illness admitted to ED	copeptin levels and the NEWS in the non-survivor patients group were higher than those in the survivor group [30.35 (14.20, 38.91) vs. 17.53 (13.01, 25.20), *p* = 0.001 and 9.0 (7.0–10.0) vs. 7.0 (6.0–8.0), *p* = 0.001] Copeptin > 19.78 pg/mL + NEWS > 8.5 had the highest predictive value of 30-day death [0.854 (95% CI, 0.798–0.899)]
López-Izquierdo, R. et al. 2025 [12]	prospective, multicenter, cohort study N = 687 (mean age 79)	2022–2023	ETCO2 and PI+ NEWS2 in predicting 30-day mortality in ED	The discrimation capacity of NEWS2 + ETCO2 + PI [AUC = 0.804 (95% CI: 0.745–0.863)] was superior to NEWS2 [AUC = 0.769 (95% CI: 0.707–0.831)] and to EtCO2 + PI [AUC = 0.737 (95% CI: 0.66–0.814)]
Kim, D.K. et al. 2024 [24]	Retrospective case-control, monocentric study N = 683 (>65 years)	2022	MEWS vs. NEWS in predicting in-hospital mortality in NTC geriatric patients	NEWS and MEWS of non-survivors were higher than in survivors (NEWS, 7 [5,6,7,8,9,10] vs. 10 [8,9,10,11,12]; MEWS, 5 [4,5,6] vs. 7 [5,6,7,8]). The NEWS (aOR]; 1.253, 95% CI: 1.181–1.329) and MEWS (aOR; 1.323, 95% CI: 1.206–1.451) were also significantly associated with in-hospital death. NEWS AUROC= 0.721 (95% CI: 0.685–0.754) vs. MEWS AUROC = 0.695 (95% CI: 0.659–0.730)
Mitsunaga, T. et al. 2019 [10]	retrospective, single-centre observational study N= 2204 (>65 years)	2017–2018	NEWS vs. MEWS at admission to ED in predicting in-hospital mortality	NEWS was a better predictor than MEWS (AUC 0.789 for NEWS vs. AUC 0.720 for MEWS) (*p* < 0.001)
Tapsiz, H. et al. 2023 [25].	Retrospective monocentric study N = 500 (age > 65 years)	2020–2021	NEWS2 vs. qSOFA in predicting mortality in alterated mental status geriatric patients	NEWS2 was a better predictor of mortality compared with quick-SOFA (qSOFA) [AUC 0.725 (CI: 0.683–0.763) vs. 0.631 (CI: 0.587–0.673)]
Nissen, S.K. et al. 2022 [15]	International multicenter retrospective cohort study N= 50,448 (patients of 18 to 64 years, 65 to 80 years, and >80 years)	2008–2013	NEWS for predicting mortality in different age categories	Mortality risk was underestimatied in the >80 years cohort compared with the others [AUROC 0.82 (CI 0.80 to 0.84)] Combining NEWS with age the underestimation was correct [AUROC 0.86 (CI 0.85 to 0.88)] (*p* < 0.001)
Küçükceran, K. et al. 2024 [26]	Retrospective monocentric N = 651 (>65 years)	2021	NEWS2 vs. LDT-EWS in predicting mortality	Median IQR of NEWS2 and LDT-EWS of the death patients were statistically significantly higher than in survivors (NEWS2: 5 [3,4,5,6,7,8] vs. 3 [1,2,3,4,5]); *p* < 0.001; LDT-EWS: 8 [7,8,9,10] vs. 6 [5,6,7,8]; *p* < 0.001). NEWS2 + LDT-EWS was the best predictor of mortality (AUC 0.775 vs. 0.717 of NEWS2 and 0.705 of LDT-EWS alone)
Kim, I. et al. 2020 [27]	Retrospective, observational, monocentric study N= 3169 (mean age 75 years)	2016–2017	NEWS in older people (<65 years)	NEWS was higher in non-survivors than in survivors (5 [IQR, 3–8] vs. 1 [IQR, 0–3], *p* < 0.001) AUROC 0.820 (95% CI 0.806 to 0.833) in predicting in-hospital mortality
Martín-Conty, J.L. et al. 2024 [28]	Multicenter prospective, observational study N = 8028	2019–2023	Including age in EWS (mREMS, NEWS2, ViEWS and RAPS) in people >75 years	AUC in all the scores (*p* = 0.006 for nonage-adjusted mREMS, *p* = 0.001 for NEWS2, *p* = 0.002 for ViEWS, *p* =0.028 for RAPS, all compared with their counterparts with age) NEWS2 with age showed the best performance (AUC = 0.899)
Dundar, Z.D. et al. 2020 [29]	retrospective observational noncentric study N = 455 (≥65 years, mean age 76 years)	2017	NEWS vs. Lactate vs. NEWS-L in predicting mortality in geriatric patients admitted to ED	lactate, NEWS, and NEWS-L of non-survivors was significantly higher than those of survivors (2.9 ± 2.2 vs. 1.9 ± 1.5 mmol/L, 8.9 ± 4.1 vs. 6.1 ± 3.7, and 11.8 ± 5.0 vs. 8.1 ± 4.4, respectively, for all *p* < 0.001). The AUCs of the lactate, NEWS, and NEWS-L in predicting in-hospital mortality were respectively 0.654 (95% CI 0.594–0.713), 0.686 (95% CI 0.628–0.744), and 0.714 (95% CI 0.658–0.770)
Nagae, M. et al. 2025 [30]	Retrospective monocentric cohort study N = 872 (>65 years)	2020–2023	FI-lab vs. NEWS	FI-lab showed predictive ability (AUC = 0.72, 95% CI: 0.66–0.78) as good as that of NEWS (AUC = 0.70, 95% CI 0.64–0.76)
Wu, P.H. et al. 2023 [31]	Retrospective monocentric observational study N = 336	2016–2021	RAPS vs. REMS vs. MEWS in older patients (>65 years) with acute upper gastrointestinal bleeding in ED	MEWS had the higher accuracy in predicting in-hospital mortality (AUC = 0.82) (*p* < 0.001)
Özdemir, S. et al. 2022 [32]	prospective, single-center, observational study N = 122 (>65 years)	2020	MEWS vs. REMS vs. RAPS in predicting in-hospital mortality in elderly patients with COVID 19 infection in ED	All scores are not statistically able in predicting mortality. AUC of MEWS, RAPS, and REMS were 0.512 (95% [CI]: 0.420–0.604; *p* = 0.910), 0.500 (95% CI: 0.408–0.592; *p* = 0.996), and 0.675 (95% CI: 0.585–0.757; *p* = 0.014), respectively
Dynesen, J. et al. 2019 [33]	Retrospective, observational, monocentric cohort study N = 19 123	2015	EWS (MEWS, NEWS, ViEWS) in predicting mortality in different age groups (16– 59 years, 60–79 years, 80+ years)	Oldest patients (80+ years) have a higher 7-day mortality compared to young patients (16–59 years) with a similar initial EWS
Kabell Nissen, S. et al. 2023 [34]	Single-center prospective observational, cohort study N = 2250 (>65 years)	2019	FaP-ED vs. NEWS vs. CFS in predicting 30-day mortality in geriatric patients admitted to ED	AUROC) for FaP-ED was 0.86 (95% CI = 0.83–0.90) significantly higher than NEWS (0.81, 95% CI = 0.77–0.85, *p* < 0.001) or CFS alone (0.82, 95% CI = 0.78–0.86, *p* < 0.001)
Gosselin, M. et al. 2022 [35]	monocentric prospective study N = 602 (>65 years)	2019–2020	Lactate vs. MEWS and MEWS in predicting poor outcomes in geriatric patients admitted to ED	Lactate was not significantly associated with poor outcome (OR = 1.12, *p* = 0.31 MEWS was signifcantly associated with poor outcome (OR = 4.34; *p* < 0.01; AUC = 0.54) NEWS was significantly associated with poor outcome (OR = 2.17; *p* < 0.02; AUC = 0.58). The combination of lactate level and MEWS signifcantly predicted poor outcome (OR = 4.17; *p* < 0.01; AUC = 0.58), but with a lower OR than MEWS alone
Thorén, A. et al. 2024 [36]	Prospective, multi-centre study N = 917 (mean age 72)	2019–2020	NEWS2 vs. NEWS2 plus age in predicting 30-day mortality in geriatric patients identified by RRT	NEWS2 + age has the best predictive value (AUROC 0.66 of NEWS2, 0.62–0.70 vs. 0.70 of NEWS2 + age, 0.65–0.73, *p* = 0.01, 95% CI)
Arjan, K. et al. 2021 [37]	Prospective multicentric cohort study N = 8974 (>65 years)	2017–2018	OPERA VS NEWS2 AND CFS in predicting mortality	OPERA was beter than other single scores in predicting mortality [AUROC was 0.79 (95% CI 0.78–0.80), superior to NEWS2 0.65 (0.62–0.67) and CFS 0.76 (0.74–0.77) (*p* < 0.0001).]
Cetınkaya, H.B. et al. 2017 [38]	Retrospective monocentric study N = 616 (<65 years)	2015	ViEWS-L in predicting mortality	ViEWS.L performed better that VIEWS in predicting mortality [AUROC 0.872 (*p* < 0.001)] Every unit increase in the ViEWS-L score increases the mortality risk 1.286 times (95% CI: 1.185–1.396)
Akman, C. et al. 2022 [39]	Retrospective, cross-sectional, single-center study N = 480 (<65 years)	2020–2021	NEWS vs. qSOFA, CCI, ECI in predicting mortality in. COVID-19 patients	Non-survivors had higher NEWS, q-SOFA, CCI, and ECI scores (*p* = 0.001 for NEWS, *p* < 0.001, for all others) qSOFA performed better that other scores [OR 4.276 (3.091–5.914) (*p* < 0.001) vs. NEWS OR 1.131 (1.054–1.215)]
Hori, T. et al. 2024 [40]	Retrospective, single-center study N = 260 (>65 years, mean age 86 year)	2022–2023	RASI vs. NEWS2 in predicting mortality in elderly patients	RASI was higher in patients with elevated NEWS2 (*p* < 0.001) RASI AUC of 0.80 (95% CI: 0.73–0.87) in predicting 7-days mortality RASI AUC of 0.73 (95% CI: 0.66–0.81) in predicting 30-day mortality
Jurado-Palomo, J. et al. 2025 [41].	prospective, multicenter study N = 6401 (>18 years old)	2019–2022	PDDS vs. NEWS2, REMS, RAPS in predicting mortality	No significance difference between PDDS and other scores (*p* < 0.001) AUROC of PDDS was 0.86 (95% CI: 0.816–0.903) versus NEWS2 0.866 (95% CI: 0.822–0.911), *p* = 0.828; versus REMS 0.885 (95% CI: 0.845–0.924), *p* = 0.311; versus RAPS 0.886 (95% CI: 0.846– 0.926), *p* = 0.335, respectively.
Mitsunaga, T. 2023 [42]	single-center prospective pilot study N = 47 (>70 years)	2020–2022	suPAR vs. suPAR-NEWS in predicting mortality	suPAR AUC [0.805 (95% CI 0.633–0.949, *p* < 0.001)] was almost the same as that of NEWS [0.816 (95% CI 0.668–0.934, *p* < 0.001)]. suPAR AUC [0.805 (95% CI 0.633–0.949, *p* < 0.001)] was lower than suPAR-NEWS AUC [0.865 (95% CI 0.747–0.958, *p* < 0.001)].
Zelis, N. et al. 2020 [43]	Prospective multi-centre cohort study N = 792 (<65 years, mean age 79 years)	2016–2017	RISE UP score vs. MEWS	RISE UP score had better performance than MEWS in predicting mortality [AUC 0.83 (95% CI = 0.78–0.87 vs. AUC 0.57 (0.47–0.66)]
Covino, M. et al. 2021 [44]	single-center, retrospective observational stud N = 210 (>60 years, mean age 74 years)	2020	NEWS vs. ISARIC-4C, COVID-GRAM, qCSI in predicting mortality in COVID 19 elderly patients	NEWS had similar performance than other scores [AUROC 0.764 (0.700–0.819), *p* < 0.001, vs. 0.799 (0.738–0.851 for the ISARIC-4C, 0.785 (0.723–0.838) for the COVID GRAM, 0.749 (0.685–0.806) for the qCSI)

Abbreviations: NEWS/NEWS2 = National Early Warning Score (2); ED = Emergency Department; AUC = area under the curve; CI = Confidence Interval; IQR = Interquartile Range; aOR = adjusted odds ratio; IEWS = International Early Warning Score; MEWS = Modified Early Warning Score; ICU = Intensive Care Unit; LDT-EWS = Laboratory Data Decision Tree Early Warning Score; ROSS = Ramathibodi Older Sepsis Score; ETCO2 = end-tidal carbon dioxide; PI = Perfusion Index; qSOFA = Quick Sequential Organ Failure Assessment; REMS = Rapid Emergency Medicine Score; RAPS = Rapid Acute Physiology Score; OPERA = Older Persons’ Emergency Risk Assessment; PDDS = Prehospital Drug-Derived Score; NTC = Non-Traumatic Coma; ViEWS = VitalPac Early Warning Score; Fi-lab = Frailty Index–Laboratory; CFS = Clinical Frailty Scale; FaP-ED = Frailty-adjusted Prognosis in the Emergency Department; CCI = Charlson Comorbidity Index; ECI = Elixhauser Comorbidity Index; RASI = Respiratory Adjusted Shock Index; RRT = Rapid response Team; suPAR = Soluble urokinase plasminogen activator receptor; ISARIC-4C = International Severe Acute Respiratory Infection Consortium Clinical Characterization Protocol Coronavirus Clinical Characterization Consortium; COVID GRAM = COVID-GRAM Critical Illness Risk Score; qCSI = quick COVID-19 Severity Index.

**Table 2:** Clinical trials among the role of EWSs in predicting ICU admission in geriatric patients in the ED.

Wang, T.H. et al. 2021 [45]	Retrospective monocentric cohort study N = 409 >65 years (mean age 79.5)	2010–2015	Elderly patient with influenza admitted to ED	NEWS ≥ 8 predicted ICU admission with an OR of 5.37 (95% CI 2.61 to 11.04)
Kao, C.C. et al. 2021 [46]	retrospective monocentric cohort study N = 1423 (≥65 years old)	2018–2019	Elderly patients admitted to ICU	EDMEWS progression was highly correlated with 30-day mortality (*p* < 0.001, OR = 1.607, 95% CI = 1.163–2.223)
Bech, L.K. et al. 2020 [47]	prospective observational monocentric study N = 109 (61 frail, 48 non-frail)	2017–2018	Elderly patients with NEWS ≥ 7	10 of the 61 frail patients (16%) admitted to ICU compared to 9 of the 48 non-frail patients (19%), [(aOR) 0.92 (95% CI 0.32–2.62)] Frail patients were more likely to have new treatment restrictions (aOR 2.91; 95% CI 1.26–6.71). Frail patients had a higher mortality rate (46% vs. 27%) [aOR 1.95 (95% CI 0.84–4.55)]
Dundar, Z.D. et al. 2016 [48].	prospective, single-centered observational study N= 671 (>65 years)	2014	MEWS vs. VIEWS	Both MEWS and VIEWS are effective in predicting ICU admission [AUC 0.727 and 0.756 (95% CI 0.720–0.792) respectively] (*p* < 0.001) Both MEWS and VIEWS are effective in predicting in-hospital mortality [AUC 0.891 and 0.900 (95% CI 0.860–0.941) respectively] (*p* < 0.001) No statistical significant differences were found between 2 scores
Kim, S.H. et al. 2021 [49]	Single-center, retrospective cohort study N = 2814	2019–2020	NEWS in patients identified by RRT	No statistically differences in patients <80 and >80 years old for ICU admission (AUROC 0.639 [95% CI 0.601–0.677] vs. 0.666 [0.614–0.718], *p* = 0.409), for ICU mortality (AUROC 0.560 [95% CI 0.444–0.675] vs. 0.582 [0.478–0.687], *p* = 0.777) and for in-hospital mortality (AUROC 0.666 [95% CI 0.642–0.691] vs. 0.648 [0.615–0.680], *p* = 0.365)
Lv, C. et al. 2021 [50]	Retrospective single-center study N = 1044 (>65 years)	2018–2020	MEWS and NEWS vs. CURB65 and qSOFA in predicting ICU admission and mortality in older adults with pneumonia	MEWS was superior in predicting mortality [AUC 0.927 (*p* < 0.001) vs. 0.844 (*p* < 0.001) for qSOFA, 0.868 (*p* < 0.001) for CURB65, and 0.892 (*p* < 0.001) for NEWS] NEWS was superior in predicting ICU admission [AUC 0.976 (*p* < 0.001) vs. 0.866 (*p* < 0.001) for qSOFA, 0.854 (*p* < 0.001) for CURB-65 and 0.922 (*p* < 0.001) for MEWS]
Bae, S.J. et al. 2025 [51]	retrospective observational monocentric case-control study N= 3757 (18–64 years) and N= 3174 (>65 years)	2023	NEWS2, and REMS in predicting ICU admission and in-hospital mortality in older patients (>65 years) vs. younger patients (18–64 years)	REMS showed significantly lower AUROC values in older patients than adults for ICU admission (0.770 vs. 0.687, *p* < 0.001). REMS and NEWS2 showed significantly lower AUROC values in older patients for in-hospital mortality (REMS: 0.801 vs. 0.734, *p* < 0.001; NEWS2: 0.842 vs. 0.778, *p* < 0.001).

## Data Availability

No new data were created or analyzed in this study.
